# Obesity is independently associated with septic shock, renal complications, and mortality in a multiracial patient cohort hospitalized with COVID-19

**DOI:** 10.1371/journal.pone.0255811

**Published:** 2021-08-12

**Authors:** Gabrielle Page-Wilson, Rachel Arakawa, Samantha Nemeth, Fletcher Bell, Zachary Girvin, Mary-Claire Tuohy, Max Lauring, Blandine Laferrère, Gissette Reyes-Soffer, Karthik Natarajan, RuiJun Chen, Paul Kurlansky, Judith Korner

**Affiliations:** 1 Division of Endocrinology, Diabetes, & Metabolism, Department of Medicine, College of Physicians and Surgeons, Columbia University Irving Medical Center, New York, New York, United States of America; 2 Division of Endocrinology, Diabetes, and Bone disease, Icahn School of Medicine at Mount Sinai, New York, New York, United States of America; 3 Columbia HeartSource, Center for Innovation and Outcomes Research, Columbia University Irving Medical Center, New York, New York, United States of America; 4 Vagelos College of Physicians and Surgeons, Columbia University Irving Medical Center, New York, New York, United States of America; 5 Division of Preventive Medicine and Nutrition, College of Physicians and Surgeons, Columbia University Irving Medical Center, New York, New York, United States of America; 6 Department of Biomedical Informatics, College of Physicians and Surgeons, Columbia University Irving Medical Center, New York, New York, United States of America; 7 Translational Data Science and Informatics, Geisinger Health, New York, New York, United States of America; Osaka University Graduate School of Medicine, JAPAN

## Abstract

**Background:**

Obesity has emerged as a risk factor for severe coronavirus disease 2019 (COVID-19) infection. To inform treatment considerations the relationship between obesity and COVID-19 complications and the influence of race, ethnicity, and socioeconomic factors deserves continued attention.

**Objective:**

To determine if obesity is an independent risk factor for severe COVID-19 complications and mortality and examine the relationship between BMI, race, ethnicity, distressed community index and COVID-19 complications and mortality.

**Methods:**

A retrospective cohort study of 1,019 SARS-CoV-2 positive adult admitted to an academic medical center (n = 928) and its affiliated community hospital (n-91) in New York City from March 1 to April 18, 2020.

**Results:**

Median age was 64 years (IQR 52–75), 58.7% were men, 23.0% were Black, and 52.8% were Hispanic. The prevalence of overweight and obesity was 75.2%; median BMI was 28.5 kg/m^2^ (25.1–33.0). Over the study period 23.7% patients died, 27.3% required invasive mechanical ventilation, 22.7% developed septic shock, and 9.1% required renal replacement therapy (RRT). In the multivariable logistic regression model, BMI was associated with complications including intubation (Odds Ratio [OR]1.03, 95% Confidence Interval [CI]1.01–1.05), septic shock (OR 1.04, CI 1.01–1.06), and RRT (OR1.07, CI 1.04–1.10), and mortality (OR 1.04, CI 1.01–1.06). The odds of death were highest among those with BMI ≥ 40 kg/m^2^ (OR 2.05, CI 1.04–4.04). Mortality did not differ by race, ethnicity, or socioeconomic distress score, though Black and Asian patients were more likely to require RRT.

**Conclusions and relevance:**

Severe complications of COVID-19 and death are more likely in patients with obesity, independent of age and comorbidities. While race, ethnicity, and socioeconomic status did not impact COVID-19 related mortality, Black and Asian patients were more likely to require RRT. The presence of obesity, and in some instances race, should inform resource allocation and risk stratification in patients hospitalized with COVID-19.

## Introduction

Severe acute respiratory syndrome coronavirus 2 (SARS-CoV-2) is the novel coronavirus responsible for causing coronavirus disease 2019 (COVID-19). While vaccination efforts are underway, population immunity will take time and novel variants of the SARS-CoV-2 virus are emerging throughout the world. As of July 2021, there have been over 33.5 million total cases and upwards of 600,000 deaths in the United States alone [1, 2]. Obesity is increasingly characterized as a risk factor for COVID-19 infection, hospitalization, and mortality [3–7]. A recent meta-analysis capturing 399,451 patients worldwide, showed that patients with obesity were 113% more likely to be hospitalized and 48% more likely to die from COVID-19 than their counterparts with body mass index (BMI) in the normal range [7]. Additionally emerging data suggests pandemic related lockdown conditions may further contribute to the prevalence of obesity by curtailing physical activity, triggering stress eating, and making it harder to achieve weight loss goals [8, 9]. Given 40% of American adults aged 20 years and over have obesity, understanding the relationship between obesity and severe COVID-19 is of tremendous importance [10].

Among patients with obesity, adverse outcomes may be driven, in part, by comorbid conditions such as diabetes, hypertension, cardiovascular and lung disease. Outcomes may also be influenced by higher rates of obesity among populations disproportionately impacted by COVID-19, including African-American and Latinx communities, and individuals from lower income brackets. Comorbid conditions and risk factors may confound the relationship between obesity and COVID-19 outcomes, and the most informative analyses should adjust for them. However, in the early months of the COVID-19 pandemic, due to the pressing need for rapid information sharing, studies examining the relationship between obesity and COVID-19 related complications and outcomes commonly deferred statistical adjustments for comorbid conditions and risk factors, and analyses were limited by incomplete capture of BMI and terminal outcome data [11–13]. While initial reports of hospitalized patients from our institution did demonstrate a preponderance of obesity and high rates of intubation, shock, acute kidney injury, and hemodialysis [14, 15], clinical outcome assessments were curtailed because up to 37% of patients were still hospitalized at the time of data analyses [14, 15]. Reports on obesity were also limited by missing or implausible body mass index data in up to 28% of patients [16].

This study expands upon earlier retrospective reports from our quaternary academic medical center in New York City, utilizing an enriched manually validated dataset that includes complete BMI data and terminal outcome data in 99% of hospitalized patients admitted from March 1 through April 18, 2020. The goals of this report are to comprehensively examine the independent association between obesity and mortality and to investigate the relationship between obesity and severe complications of COVID-19 in a hospitalized cohort, given few studies explore the relationship between obesity and non-respiratory complications [17, 18]. Whether obesity may independently mediate adverse outcomes among population sub-groups also remains unclear [10]. The secondary objective is therefore to determine if race, ethnicity, or socioeconomic distress are associated with severe COVID-19 outcomes and mortality in our highly diverse cohort, and whether obesity may mediate severe outcomes among distinct racial/ethnic and socioeconomic groups.

## Methods

### Study design and participants

**Fig 1:**
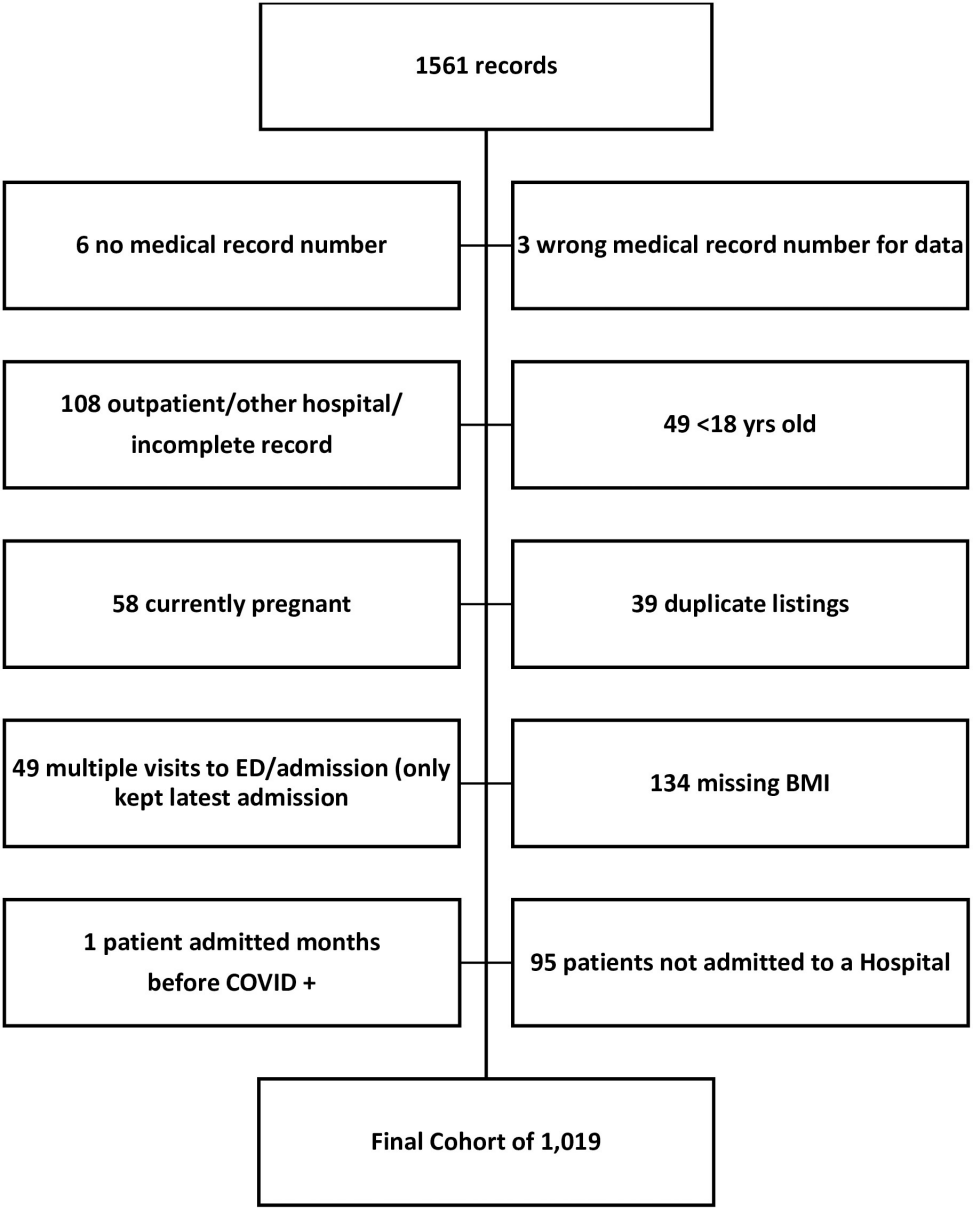
Consort diagram.

The electronic health record (EHR) and clinical data warehouse were reviewed for adult patients (age ≥ 18 years) admitted for care at two New York-Presbyterian (NYP) hospitals affiliated with Columbia University Irving Medical Center (CUIMC) between March 1 and April 18, 2020, with laboratory confirmed COVID-19 infection as demonstrated by a positive result on the SARS-CoV-2-RT-PCR test These hospitals represent a quaternary referral center (NYP/CUIMC) and community hospital (NYP/Allen), that predominantly admitted patients from surrounding neighborhoods in Northern Manhattan and the Bronx during the time period of relevance. Patient demographics, baseline physical characteristics, past medical history, medications, comorbidities, presenting symptoms, and hospital course including admission to ICU, mechanical ventilation, complications, and disposition status, were abstracted in an ongoing retrospective fashion as previously described [14]. Cases were excluded if they were not admitted to the hospital, pregnant, <18 years old, or had missing BMI data (Fig 1). In total, 1,019 patients were characterized, and continuous updates were collected on patient course and outcomes through July 1, 2020. There were nine patients who were still in-house upon completion of data collection.

### Data abstraction

The manual chart review survey included 274 data points on each patient and was stored in a HIPAA compliant REDCap database [19]. To ensure uniformity, a team of data extractors were trained in an online session by project members of the primary characterization study and continuously referenced a detailed instruction manual [14]. Information was inputted into REDCap using an instrument previously designed and validated with high inter-rater reliability by Cohen’s kappa coefficient [20]. Additional manual data abstraction pertaining to BMI, race, and ethnicity was performed if it had not been obtained on hospital admission or recorded in notes from the hospital course. Based on a search of notes from the historical EHR, BMI was included if it had been recorded within six months prior to the SARS-CoV-2-RT-PCR test-positive admission date. If multiple entries were discovered, preference was given to BMI values recorded closest to the day of admission. Race and ethnicity fields that appeared as unspecified or declined were further investigated by review of historical inpatient and outpatient records in the EHR.

### Study endpoints

The primary endpoint of interest was in-hospital mortality. Secondary endpoints included: intubation, septic shock, and the need to initiate renal replacement therapy (RRT).

### Data definitions

For data definitions, see S1 Table. RRT was defined as initiation of hemodialysis, peritoneal dialysis, continuous veno-venous hemofiltration (CVVH), or continuous renal replacement therapy (CRRT). Septic shock was defined as the necessity for vasopressors and/or inotropes for blood pressure support. The nine patients who were still in-house upon completion of data collection were excluded from mortality analysis but included in incidence of intubation, RRT and septic shock. The Distressed Communities Index (DCI), was calculated to estimate the effect of socioeconomic distress on outcomes [21]. DCI is a composite score of socioeconomic status based on seven component metrics by zip code, including unemployment, education level, poverty rate, median income, business growth, job growth, and housing vacancies; scores range from 0 (no distress) to 100 (severe distress). Scores range from 0 (no distress) to 100 (severe distress) and are divided into 5 quintiles as follows: prosperous—0–19.9, comfortable 20–39.9, mid-tier 40–59.9, at risk 60–79.9, and distressed 80–100. BMI (kg/m^2^) was classified in accord with Word Health Organization (WHO) definitions as follows: < 18.5 kg/m^2^ –Underweight, 18.5–24.9 kg/m^2^ –Normal weight, 25.0–29.9 kg/m^2^ –Overweight, 30.0–34.9 kg/m^2^ –Obesity (Class I), 35.0–39.9 kg/m^2^ –Obesity (Class II), >40.0 kg/m^2^ –Obesity (Class III) [22].

### Statistical analysis

*R* statistical software packages (version 3.6.1, R Foundation) were used for statistical analysis and all figures. Data are expressed as frequencies and percentages for categorical variables and were tested either by the Chi-Squared test or Fisher’s Exact test depending on size (>5). Continuous variables are expressed as either mean (SD) or median (IQR) depending on normality which was tested via QQ plots, and were compared using the t-test or Mann-Whitney test respectively in bivariate analyses and ANOVA or Kruskall-Wallis in comparisons with more than two groups. Post-hoc pairwise comparisons of outcomes across BMI groups used Bonferroni to correct for multiple comparisons and a p-value <0.005 was deemed significant. Next, all clinically relevant variables that were captured in the database were included in the multivariable logistic regression (MVLR) in order to account for possible confounding factors. These variables were tested for collinearity using the variance inflation factor and none were found to be colinear. For the new onset renal replacement therapy outcome, variables of interest and those deemed the most clinically significant were chosen to prevent overfitting. MVLR models were performed both with BMI as a categorical and as a continuous variable. For all regression results, a p-value of <0.05 was deemed significant.

### Ethical statement

This protocol (#AAAS9906) was approved by the Columbia University Irving Medical Center Institutional Review Board with waiver of patient consent on the basis of study design and the ongoing COVID-19 public health emergency.

## Results

Between March 1 and April 18, 2020, 1,561 adults presented to the NYP/CUIMC or NYP/Allen Hospital with laboratory-confirmed SARS-CoV-2-RT-PCR test by nasopharyngeal swab. We excluded 49 children (age <18 years), 58 pregnant women, 95 patients who did not meet criteria for admission, and 134 patients (8.5%) who did not have BMI data available after manual extraction from the inpatient and outpatient EHR (Fig 1). In the final cohort, the number of patients admitted to NYP/CUIMC and NYP/Allen Hospital were 928 and 91 respectively. Patient characteristics are presented in Table 1. The prevalence of obesity (BMI ≥30 kg/m^2^) was 41.2%. Patients with overweight (BMI 25–29.9 kg/m^2^) and obesity were significantly younger than those with normal BMI (BMI 18.5–24.9 kg/m^2^) (p < 0.001), such that individuals with BMI ≥40kg/m^2^ were the youngest compared to those in all other BMI classes. The prevalence of co-morbidities was high: 63% of patients had hypertension, 40% had Type 2 diabetes and 22% had pulmonary disease. A total of 261 patients (25.6%) were active or former smokers. The median DCI score was 74.0 (56.8–80.3) with 722 (70.8%) in the top two quintiles of socioeconomic distress, classified as “at risk” and “distressed” respectively. Notably only 38 (3.7%) of patients had DCI scores within the lowest quintile, defined as “prosperous.” There were no significant differences in ethnicity, race, or DCI scores across all BMI categories.

**Table 1:** Patient characteristics by BMI categories.

Variable	ALL	BMI <18.5	BMI 18.5–24.9	BMI 25–29.9	BMI 30–39.9	BMI ≥40	P-value
N	1019	25 (2.5)	227 (22.3)	347 (34.0)	340 (33.3)	80 (7.9)	
Age	64.0 [52.0–75.0]	75.0 [48.0–83.0]	71.0 [59.0–81.5]	65.0 [54.0–75.5]	60.0 [50.0–71.0]	52.0 [40.0–65.3]	<0.001
Male	598 (58.7)	14 (56.0)	128 (56.4)	242 (69.7)	173 (50.9)	41 (51.2)	<0.001
BMI	28.5 [25.1–33.0]	17.3 [16.0–18.0]	22.7 [21.5–24.1]	27.5 [26.1–28.6]	33.1 [31.5–35.4]	44.5 [42.2–48.8]	<0.001
Race							0.074
White	244 (23.9)	4 (16.0)	62 (27.3)	82 (23.6)	79 (23.2)	17 (21.2)	
Black	234 (23.0)	9 (36.0)	46 (20.3)	67 (19.3)	87 (25.6)	25 (31.2)	
Asian	21 (2.1)	0 (0.0)	11 (4.8)	5 (1.4)	5 (1.5)	0 (0.0)	
Other	349 (34.2)	7 (28.0)	73 (32.2)	125 (36.0)	121 (35.6)	23 (28.7)	
Not Specified	171 (16.8)	5 (20.0)	35 (15.4)	68 (19.6)	48 (14.1)	15 (18.8)	
Ethnicity							0.046
Hispanic	538 (52.8)	9 (36.0)	110 (48.5)	190 (54.8)	196 (57.6)	33 (41.2)	
Not Hispanic	285 (28.0)	10 (40.0)	73 (32.2)	84 (24.2)	89 (26.2)	29 (36.2)	
Not Specified	196 (19.2)	6 (24.0)	44 (19.4)	73 (21.0)	55 (16.2)	18 (22.5)	
Smoking							0.058
Active Smoker/Vape	54 (5.3)	3 (12.0)	9 (4.0)	22 (6.3)	16 (4.7)	4 (5.0)	
Former Smoker	207 (20.3)	8 (32.0)	57 (25.1)	70 (20.2)	54 (15.9)	18 (22.5)	
No/Unknown	758 (74.4)	14 (56.0)	161 (70.9)	255 (73.5)	270 (79.4)	58 (72.5)	
Coronary Artery Disease	142 (13.9)	3 (12.0)	39 (17.2)	51 (14.7)	40 (11.8)	9 (11.2)	0.418
Heart Failure	125 (12.3)	4 (16.0)	35 (15.4)	36 (10.4)	42 (12.4)	8 (10.0)	0.394
Stroke	87 (8.5)	3 (12.0)	31 (13.7)	23 (6.6)	26 (7.6)	4 (5.0)	0.028
Type 2 Diabetes	409 (40.1)	8 (32.0)	85 (37.4)	144 (41.5)	138 (40.6)	34 (42.5)	0.762
Hypertension	647 (63.5)	16 (64.0)	154 (67.8)	216 (62.2)	211 (62.1)	50 (62.5)	0.659
Hyperlipidemia	371 (36.4)	6 (24.0)	83 (36.6)	129 (37.2)	128 (37.6)	25 (31.2)	0.575
COPD	72 (7.1)	1 (4.0)	23 (10.1)	15 (4.3)	26 (7.6)	7 (8.8)	0.074
Asthma	105 (10.3)	1 (4.0)	19 (8.4)	32 (9.2)	36 (10.6)	17 (21.2)	0.027
Obstructive Sleep Apnea	29 (2.8)	0 (0.0)	1 (0.4)	3 (0.9)	11 (3.2)	14 (17.5)	<0.001
Renal Disease	159 (15.6)	8 (32.0)	53 (23.3)	50 (14.4)	39 (11.5)	9 (11.2)	<0.001
HIV	24 (2.4)	1 (4.0)	7 (3.1)	7 (2.0)	8 (2.4)	1 (1.2)	0.735
Active Cancer	69 (6.8)	4 (16.0)	29 (12.8)	19 (5.5)	14 (4.1)	3 (3.8)	<0.001
Transplant	46 (4.5)	3 (12.0)	13 (5.7)	17 (4.9)	11 (3.2)	2 (2.5)	0.180
Inhaled Steroids	56 (5.5)	1 (4.0)	16 (7.0)	14 (4.0)	18 (5.3)	7 (8.8)	0.335
Oral Steroids	65 (6.4)	5 (20.0)	20 (8.8)	21 (6.1)	10 (2.9)	9 (11.2)	0.001
Statins	403 (39.5)	5 (20.0)	96 (42.3)	143 (41.2)	135 (39.7)	24 (30.0)	0.086
Medication Count	5.0 [1.0–8.0]	6.0 [4.0–9.0]	6.0 [2.0–9.0]	4.0 [1.0–8.0]	5.0 [1.0–8.0]	3.0 [0.0–7.0]	0.003
DCI Score	74.0 [56.8–80.3]	77.2 [67.4–77.2]	73.6 [56.8–78.9]	77.2 [56.8–79.8]	73.6 [56.8–81.7]	73.7 [56.8–80.6]	0.978
0–19.9	38 (3.7)	1 (4.0)	8 (3.5)	10 (2.9)	15 (4.4)	4 (5.0)	
20–39.9	57 (5.6)	1 (4.0)	12 (5.3)	20 (5.8)	18 (5.3)	6 (7.5)	
40–59.9	202 (19.8)	3 (12.0)	47 (20.7)	73 (21.0)	63 (18.5)	16 (20.0)	
60–79.9	463 (45.4)	15 (60.0)	105 (46.3)	158 (45.5)	152 (44.7)	33 (41.2)	
80–100	259 (25.4)	5 (20.0)	55 (24.2)	86 (24.8)	92 (27.1)	21 (26.2)	

Data are presented as n (%) or Median [IQR]. BMI values are in kg/m^2^. COPD = chronic obstructive pulmonary disease.

Clinical outcomes are presented in Table 2. Over the study period a total of 23.7% patients died, 27.3% required invasive mechanical ventilation, 22.7% developed septic shock, and 9.1% required RRT. The median length of ICU time was 14.2 days (5.3–30.4) and 9 patients (1%) remained hospitalized at the end of the study period.

**Table 2:** Patient outcomes by BMI categories.

Variable	ALL	BMI <18.5	BMI 18.5–24.9	BMI 25–29.9	BMI 30–39.9	BMI ≥40	P-Value
Mortality	N = 1010	N = 25	N = 226	N = 343	N = 337	N = 79	**0.025**
	239 (23.7)	**6 (24.0)**	**71 (31.4)**	**72 (21.0)**	**69 (20.5)**	**21 (26.6)**	
Admitted to ICU	N = 1018	N = 25	N = 227	N = 346	N = 340	N = 80	**0.043**
	294 (28.9)	**3 (12.0)**	**57 (25.1)**	**97 (28.0)**	**106 (31.2)**	**31 (38.8)**	
ICU Time (days)	N = 286	N = 3	N = 56	N = 94	N = 103	N = 30	**0.019**
	14.2 [5.3–30.4]	**4.1 [3.0–6.8]**	**8.9 [2.8–20.7]**	**17.0 [6.4–31.6]**	**17.0 [7.2–34.8]**	**12.9 [6.4–21.4]**	
Intubation	N = 1018	N = 25	N = 227	N = 346	N = 340	N = 80	**0.025**
	278 (27.3)	**3 (12.0)**	**53 (23.3)**	**90 (26.0)**	**101 (29.7)**	**31 (38.8)**	
Intubation (days)	N = 270	N = 3	N = 52	N = 87	N = 98	N = 30	**0.008**
	14.4 [6.5–33.4]	**3.7 [2.8–10.5]**	**8.4 [2.8–22.3]**	**18.0 [9.4–38.4]**	**16.8 [8.9–34.6]**	**13.1 [6.7–20.9]**	
ARDS	N = 1018	N = 25	N = 227	N = 346	N = 340	N = 80	0.087
	369 (36.2)	**4 (16.0)**	**80 (35.2)**	**126 (36.4)**	**122 (35.9)**	**37 (46.2)**	
Septic Shock	N = 1018	N = 25	N = 227	N = 346	N = 340	N = 80	**0.027**
	231 (22.7)	**5 (20.0)**	**37 (16.3)**	**76 (22.0)**	**88 (25.9)**	**25 (31.2)**	
Acute Kidney Injury	N = 1019	N = 25	N = 227	N = 347	N = 340	N = 80	0.360
	379 (37.2)	**7 (28.0)**	**75 (33.0)**	**131 (37.8)**	**131 (38.5)**	**35 (43.8)**	
New Dialysis	N = 947	N = 19	N = 204	N = 325	N = 320	N = 79	**<0.001**
	92 (9.7)	**1 (5.3)**	**10 (4.9)**	**23 (7.1)**	**43 (13.4)**	**15 (19.0)**	
Myocardial Infarction	N = 1012	N = 25	N = 226	N = 344	N = 338	N = 79	0.631
	10 (1.0)	**0 (0.0)**	**4 (1.8)**	**4 (1.2)**	**2 (0.6)**	**0 (0.0)**	
New Heart Failure	N = 1012	N = 25	N = 226	N = 345	N = 337	N = 79	0.769
	26 (2.6)	**0 (0.0)**	**8 (3.5)**	**10 (2.9)**	**7 (2.1)**	**1 (1.3)**	
Arrhythmia	N = 1012	N = 25	N = 227	N = 344	N = 337	N = 79	0.801
	92 (9.1)	**1 (4.0)**	**24 (10.6)**	**33 (9.6)**	**27 (8.0)**	**7 (8.9)**	
Rhabdomyolysis	N = 1012	N = 25	N = 226	N = 344	N = 338	N = 79	**0.023**
	12 (1.1)	**0 (0.0)**	**0 (0.0)**	**3 (0.9)**	**5 (1.5)**	**4 (5.1)**	
DKA	N = 1012	N = 25	N = 226	N = 345	N = 337	N = 79	0.938
	29 (2.9)	**1 (4.0)**	**7 (3.1)**	**9 (2.6)**	**10 (3.0)**	**2 (2.5)**	
Thrombosis	N = 1014	N = 25	N = 227	N = 346	N = 337	N = 79	0.118
	65 (6.4)	**2 (8.0)**	**9 (4.0)**	**18 (5.2)**	**29 (8.6)**	**7 (8.9)**	
Total LOS (days)	N = 1010	N = 25	N = 226	N = 343	N = 337	N = 79	0.077
	7.0 [4.0–15.0]	**5.0 [3.0–9.0]**	**7.0 [3.0–14.0]**	**7.0 [4.0–17.5]**	**7.0 [4.0–16.0]**	**9.0 [6.0–16.0]**	

Results are presented as n (%) or median [IQR].

**Fig 2:**
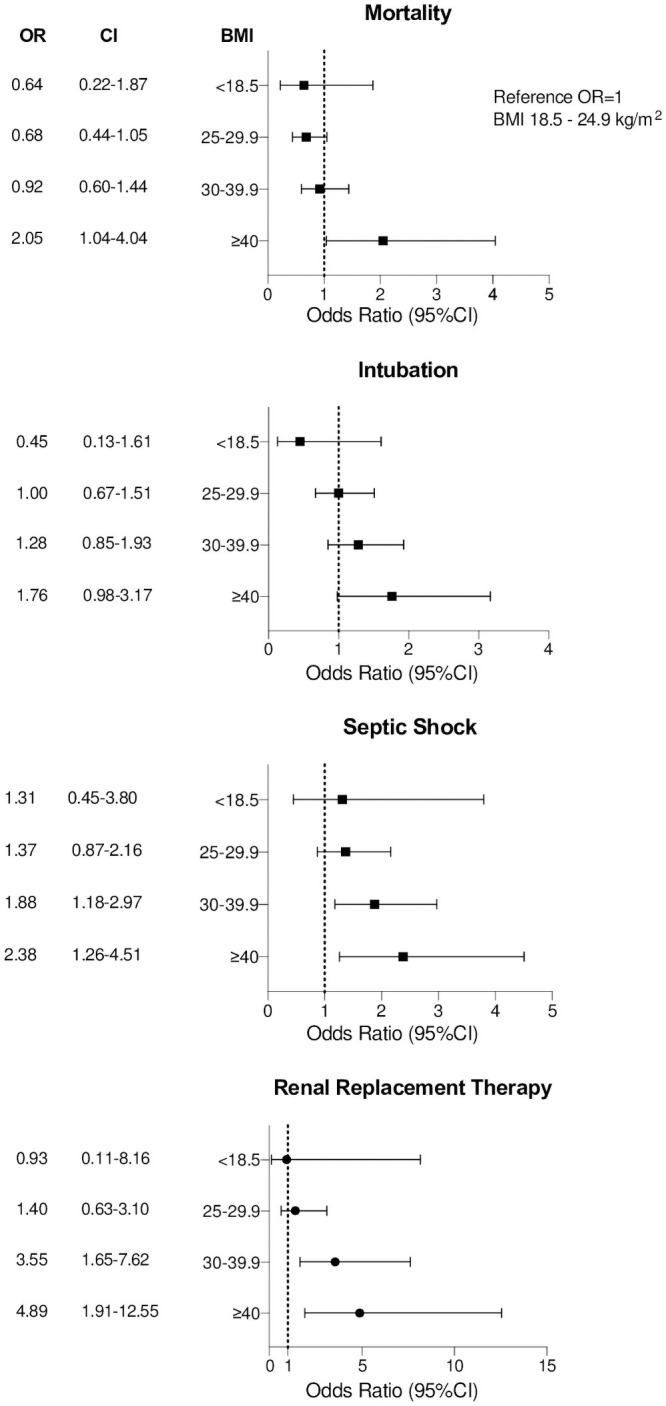
Forest plots of multivariable-adjusted associations between body mass index and end points of mortality, intubation, septic shock, and need for renal replacement therapy.

Results from univariable linear regression analysis are presented in S2 Table. In MVLR, increasing BMI was independently associated with an increased risk of death (OR 1.04, CI 1.01–1.06) (Table 3). In the MVLR model with BMI as a categorical variable, patients with BMI ≥40kg/m^2^ had the highest odds of dying (OR 2.05, CI 1.04–4.04) (Fig 2). Older age (OR 1.05, CI 1.04–1.07), male sex (OR 1.84, CI 1.30–2.59), hypertension (OR 1.65, CI 1.06–2.58), and diabetes (OR 1.46, CI 1.03–2.06) were also independently associated with an increased risk of death. In contrast, asthma was associated with lower mortality (OR 0.48, CI 0.25–0.91). There was no difference in mortality between patients with asthma using inhaled or oral steroids prior to hospitalization compared to those who were not taking any steroids (*P* = 0.56).

**Table 3:** Multivariable regression analysis of BMI and endpoints of relevance.

	Mortality N = 239	Intubation N = 278	Septic shock N = 231	Renal Replacement Therapy N = 92
Age	1.05 (1.04–1.07)***	0.99 (0.98–1.00)	1.00 (0.99–1.01)	0.99 (0.98–1.01)
Male Sex	1.84 (1.30–2.59) **	1.86 (1.36–2.53)***	1.61 (1.16–2.23)**	3.08 (1.81–5.24)***
BMI	1.04 (1.01–1.06)**	1.03 (1.01–1.05)**	1.04 (1.01–1.06)**	1.07 (1.03–1.10)***
Race				
White (ref)				
Asian	1.95 (0.67–5.68)	1.92 (0.72–5.08)	3.03 (1.15–7.97)*	7.12 (2.05–24.70)**
Black	1.16 (0.72–1.88)	1.14 (0.75–1.75)	1.37 (0.87–2.15)	2.24 (1.15–4.37)*
Other	1.06 (0.68–1.67)	0.78 (0.52–1.17)	0.91 (0.58–1.42)	1.07 (0.53–2.18)
Not specified	1.57 (0.93–2.66)	1.03 (0.63–1.67)	1.31 (0.79–2.19)	1.47 (0.64–3.35)
Ethnicity				
Not Hispanic (ref)				
Hispanic	1.03 (0.67–1.59)	0.99 (0.68–1.45)	0.80 (0.54–1.20)	0.74 (0.42–1.30)
Not Specified	0.77 (0.46–1.30)	0.86 (0.54–1.37)	0.93 (0.58–1.50)	0.50 (0.24–1.05)
Hypertension	1.65 (1.06–2.58)*	1.86 (1.27–2.73)**	1.78 (1.19–2.68)**	1.54 (0.87–2.75)
Diabetes	1.46 (1.03–2.06)*	1.16 (0.84–1.59)	1.17 (0.84–1.64)	1.10 (0.67–1.80)
Asthma	0.46 (0.24–0.89)*	1.12 (0.70–1.78)	0.77 (0.46–1.31)	-
COPD	1.51 (0.86–2.64)	0.94 (0.52–1.71)	0.75 (0.39–1.45)	-
Coronary artery disease	1.04 (0.66–1.65)	0.83 (0.52–1.33)	0.82 (0.50–1.36)	-
Heart failure	1.21 (0.75–1.94)	0.91 (0.55–1.48)	1.08 (0.65–1.80)	-
Stroke	1.40 (0.85–2.33)	0.87 (0.50–1.52)	0.76 (0.41–1.38)	-
Hyperlipidemia	0.87 (0.59–1.26)	0.93 (0.65–1.33)	0.96 (0.66–1.41)	-
Renal Disease	1.06 (0.69–1.63)	0.65 (0.41–1.00)	0.58 (0.36–0.93)*	1.53 (0.75–3.13)
Active Cancer	1.84 (1.02–3.34)*	0.82 (0.44–1.52)	1.03 (0.56–1.91)	-
Statins	0.83 (0.56–1.23)	1.01 (0.70–1.47)	1.04 (0.70–1.54)	-

*P<0.05;

**P<0.01;

***P<0.001.

Patient characteristics independently associated with the need for intubation were BMI (OR 1.03, CI 1.01–1.05), male sex (OR 1.86, CI 1.36–2.53), and hypertension (OR 1.86, CI 1.27–2.73). Patient factors independently associated with RRT were BMI (OR 1.07, CI 1.04–1.10) and male sex (OR 2.97, Ci 1.75–5.02). When BMI was analyzed as a categorical variable, the likelihood of needing RRT increased with progressively higher BMI such that a greater than 3-fold increase was observed for BMI ≥30 (OR 3.9, CI 1.82–8.33) and greater than 5-fold increase was observed for BMI ≥40 (OR 5.95, CI 2.34–15.11) compared to normal weight patients. Notably, Black (OR 2.22, CI 1.14–4.30) and Asian race (OR 6.31, CI 1.82–21.87) increased the likelihood of requiring RRT in the hospital.

In MVLR with BMI as a continuous variable, independent patient factors associated with the development of septic shock were BMI (OR 1.04, CI 1.01–1.06), male sex (OR 1.61, CI 1.16–2.23), and hypertension (OR 1.78, CI 1.19–2.68) (Table 3). In MVLR with BMI as a categorical variable, there was a higher odds of developing septic shock at BMI 30–39.9 (OR 1.88, CI 1.18–2.97) and ≥40 kg/m^2^(OR 2.38, CI 1.26–4.51) compared to normal weight (Fig 2). Notably, Asian race (OR 3.13, CI 1.18–8.31) was also associated with the development of shock.

## Discussion

We found that obesity is independently associated with an increased risk for septic shock, renal replacement therapy, intubation and death in adults hospitalized for COVID-19 in an academic medical center at the height of the pandemic in New York City. By utilizing a manually validated and comprehensive data set, this report improves upon earlier reports that are limited by incomplete BMI and terminal outcome data. Furthermore, our study adds to the existing literature by identifying severe non-respiratory COVID-19 complications that are associated with obesity, independent of age, sex, race/ethnicity, and multiple comorbid conditions. It is noteworthy that the prevalence of individuals with obesity in our cohort was 41%, while the prevalence of obesity in the surrounding neighborhood of Northern Manhattan is approximately 26% [23]. This observation parallels data from a study of nearly 17,000 patients hospitalized with COVID-19, in which the prevalence of obesity was 48%, underscoring the prominent role that obesity plays in COVID-19 disease severity [24].

While there are some discrepancies in the literature, the observed association between obesity and COVID-19 related mortality is consistent with the findings of several other studies [3, 6, 16, 20, 25, 26]. Our findings as they relate to mortality, are also consistent with a recent study by Tartof et. al., that better captures BMI and adjusts for co-morbidities and obesity related risk factors [25]. Some authors have suggested that higher BMI is strongly associated with mortality in younger adults, and as such did not consistently observe an association between obesity and mortality in older adults [4, 16]. We found no significant interaction between age and BMI (*P* = 0.08).

Large body mass, neck and waist circumferences can increase the risk of hypoventilation and complicate supportive treatments including mask ventilation, intubation, and prone positioning. However, additional mechanistic pathways have been proposed to explain how excess adiposity alone may contribute to complications of COVID-19 [27, 28]. High ACE2 receptor expression in adipose tissue may facilitate coronavirus entry and prolonged shedding. Ectopic fat and proinflammatory immune cells that accumulate in adipocytes might impair innate and adaptive immune responses to infection and delay viral clearance, as previously demonstrated in obese individuals infected with H1N1 Influenza A [7, 29, 30]. In COVID-19, adiposity is associated with a preponderance of pro-inflammatory cells in hypertrophic adipocytes, that contribute to increases in serum cytokines, such as IL-6, TNF-alpha and CRP [7, 31]. The accumulation of adipocytes may therefore precipitate immune activation and cytokine production during COVID-19 infections, contributing to the severity of infection [7]. Inflammatory actions of adipocytes may also contribute to extrapulmonary complications of SARS- CoV2 [32].

Accordingly, we found that obesity is independently associated with COVID-19 induced septic shock. Despite frequent reports of septic shock as a severe complication of SARS Co-V2 infection, to our knowledge, only one other cohort study has examined its association with obesity [13, 18, 33]. Onder et al. found that obesity independently increased the probability of septic shock in patients hospitalized with COVID-19 in Italy, although the multivariate model did not specifically control for obesity related comorbidities. In addition, our study is the first to report a striking increase in the odds of RRT in patients with obesity independent of comorbidities, with the odds increasing by 3-fold in those with a BMI ≥30, and 5-fold in patients with morbid obesity. While an association between obesity and acute renal failure in COVID-19 has been reported [18], the study did not control for the potential confounding effects of diabetes and hypertension. Nonetheless, increased BMI may contribute to renal injury through inflammatory pathways that act independently of coexisting hypertension and diabetes [34]. Additionally, obesity related pro-inflammatory response to SARS-CoV2, could account for the stepwise increase in RRT with increasing BMI.

Consistent with the Center for Disease Control guidance [35] male sex, older age, diabetes, and hypertension were also associated with increased mortality. In contrast to other reports [25], in our cohort, hyperlipidemia and cardiovascular disease did not increase the odds of death, and hypertension was the only comorbidity that significantly increased the risk of intubation, septic shock, and RRT. Despite early concerns that asthma was a risk factor for adverse outcomes from COVID-19, asthma was associated with lower mortality among hospitalized patients in our cohort. Consistent with our findings, several recent studies have found no differences in the risk of severe COVID-19 among patients with and without asthma [36–38]. Additionally, a large meta-analysis of 410, 382 patients by Liu et al., also observed a lower risk of death among those with asthma as compared to those without [39]. This risk reduction may be attributed, in part, to lower interferon levels in patients with asthma—which could attenuate the cytokine storm that occurs in severe COVID-19, or to the downregulation of angiotensin-converting-enzyme-2 (ACE-2) receptors (one of the binding sites for SARS-CoV-2) in patients with T2-high asthma [40]. Inhaled corticosteroids, which lower ACE-2 expression [41] and are mainstay of asthma therapy, may also contribute to the observed risk reduction [40], however corticosteroid use did not impact mortality in our cohort.

Despite the fact that race, ethnicity, and socioeconomic status are known risk factors for obesity, we did not observe significant differences in these social determinants of health across BMI categories. Similarly, a prospective study conducted in Scotland did not find a difference in mortality or complications in hospitalized patients with COVID-19 with higher distress scores based on the Scottish Index for Multiple Deprivation (SIMD) [42, 43]. Although there is a disproportionate burden of COVID-19 related outcomes among Black and Latinx individuals throughout the United States [6, 44–54], like ours, the plurality of retrospective cohort studies have not demonstrated significant associations between race, ethnicity, and hospital mortality. Notably, outcomes comparisons were not made among those with COVID-19 who were not admitted to the hospital. Our study shares this limitation. The majority of hospitalized patients in our cohort came from distressed communities, as evidenced by high median DCI scores, which may be ameliorating race-based differences in mortality. While 23.7% of patients died during the study period, this startling number is similar to mortality outcomes in other academic hospitals serving demographically similar patient populations with high rates of comorbidities in New York City during the early stages of the pandemic [55].

We did find racial differences in the odds of severe extra-pulmonary complications of COVID-19. Black patients were more than twice as likely to require RRT than their white counterparts. African-American race has been associated with higher rates of AKI and need for RRT in hospitalized patients with and without COVID-19 [56–59]. Genetic factors may also be contributory, as the *APOL1* high risk genotype was found in a case series of African-American patients with COVID-19 who developed collapsing glomerulopathy and the need for RRT [60]. Asian patients were more than six times more likely to need RRT and were 3 times more likely to develop septic shock, although the number of patients was small.

This study has limitations which should be acknowledged. First, the data presented is from a single center in New York City, which may limit its generalizability as this early cohort may be epidemioloigcally distinct from populations that have subseqeuntly been impacted by the pandemic. Second, while every effort was made to manually validate all parameters, the data is not exempt from the inherent shortcomings of retrospective chart reviews and although more complete than other data sets there is still potential for residual confounding. Additionally, because BMI was critical to our analysis, we excluded patients for whom it was unavailable and the potential for selection bias therefore exists. While the study included a sizable overall cohort, the small number of patients in some racial categories may limit subgroup analyses. It should also be noted that because the study included patients admitted early in the pandemic, during a time when clincal interventions and practice patterns were in a dynamic state and disaster conditions were present due patient volume, outcomes may differ from those currently observed.

## Conclusions

Our study affirms obesity as a risk factor for severe complications of COVID-19 and death, independent of age, sex, race, ethnicity, socioeconomic distress, and comorbidities. We add to the literature by analyzing a validated cohort with terminal outcome data for almost all patients. While obesity does not seem to be mediating mortality outcomes in racial and ethnic minorities disproportionately impacted by COVID-19, racial differences in severe extra-pulmonary complications are present and accordingly both BMI and race are central to considerations of risk stratification and resource allocation.

## Supporting information

S1 Table Data definitions for parameters included in Table 1. (DOCX)

S2 Table Data definitions for parameters included in Table 2. (DOCX)

S3 Table Univariable logistic regression of BMI and relevant outcomes including a) intubation, b) mortality, c) septic shock, d) renal replacement therapy. (DOCX)

S1 Dataset (XLSX)
