# Supplementary material for: Obesity is independently associated with septic shock, renal complications, and mortality in a multiracial patient cohort hospitalized with COVID-19
Source: PLoS One. 2021 Aug 12;16(8):e0255811. doi: 10.1371/journal.pone.0255811 (PMC8360607; doi:10.1371/journal.pone.0255811)
Supplement: S1 Table — (DOCX) [file pone.0255811.s001.docx]

**S1 Table. Data definitions for parameters included in Tables 1 and 2.**

| **Patient Characteristics Prior to Hospitalization** | **Definition** |
| --- | --- |
| Smoking Status | Documentation of smoking history in patient’s chart as past medical history. |
| Coronary Artery Disease | Documentation of coronary artery disease or previous myocardial infarction in the patient’s chart as past medical history. |
| Heart Failure | Documentation of congestive heart failure in the patient’s chart as past medical history. |
| Stroke | Documentation of a prior cerebrovascular accident in the patient’s chart as past medical history. |
| Diabetes | Documentation of type 1 diabetes mellitus or type 2 diabetes mellitus in the patient’s chart as past medical history. |
| Hypertension | Documentation of hypertension in the patient’s chart as past medical history. |
| Hyperlipidemia | Documentation of hyperlipidemia in the patient’s chart as past medical history. |
| COPD | Documentation of COPD in the patient’s chart as past medical history. |
| Asthma | Documentation of a history of asthma in the patient’s chart as past medical history. |
| Obstructive Sleep Apnea | Documentation of obstructive sleep apnea in patient’s chart as past medical history. |
| Renal Disease | Documentation of chronic kidney disease (CKD), end stage renal disease (ESRD), or a baseline creatinine greater than 2.0 in the patient’s chart as past medical history. |
| HIV | Documentation of history of HIV in patient’s chart as past medical history. |
| Active Cancer | Documentation in the patient’s chart of a solid or liquid tumor that was either:  1. diagnosed within six months of admission  2. receiving cancer therapy at time of admission  3. recurrent or metastatic.  Non-melanoma skin cancer was excluded. |
| Transplant | Documentation of any history of transplant in patient’s chart including   1. Bone Marrow 2. Kidney 3. Liver 4. Heart   Methods for transplantation included deceased donor, living donor and orthotopic. |
| Inhaled Steroids | Documentation of at-home use of Fluticasone or equivalent inhaled steroid in the patient’s chart. |
| Oral Steroids | Documentation of at-home use of either prednisone, hydrocortisone, dexamethasone, budesonide, or equivalent oral steroid in the patient’s chart. |
| Statins | Statin listed as an active medication at the time of admission in the patient’s EHR. |
| Medication Count | Number of prescription medications the patient took at home prior to hospitalization as documented in the patient’s chart. |
| DCI - Distressed Community Index ^22^ | Calculated by using composite score based on seven component metrics by zip code, including unemployment, education level, poverty rate, median income, business growth, job growth, and housing vacancies |
