# Supplementary material for: Obesity is independently associated with septic shock, renal complications, and mortality in a multiracial patient cohort hospitalized with COVID-19
Source: PLoS One. 2021 Aug 12;16(8):e0255811. doi: 10.1371/journal.pone.0255811 (PMC8360607; doi:10.1371/journal.pone.0255811)
Supplement: S2 Table — (DOCX) [file pone.0255811.s002.docx]

**S2 Table. Definitions.**

| **Outcomes** | **Definitions** |
| --- | --- |
| Mortality | Documentation of death in chart occurring in the hospital including time and date of death. |
| ICU Admission | Documentation of admission into the intensive care unit at least once during the course of admission. |
| ICU Time | Length of time from ICU admission to ICU discharge. Total time was summed for patients with multiple ICU admissions in the same hospitalization. |
| Intubation | Documentation of intubation at least once during the course of admission. |
| Intubation Time | Length of time from documented time of intubation to documented time of extubation. Total time was summed for patients with multiple intubations in the same hospitalization. |
| ARDS | As defined by clinical notes in EHR (all met Berlin Criteria, not further categorized by severity). |
| Septic Shock | Documentation of the need to use vasopressors and/or inotropes for blood pressure support in patient’s chart. |
| Acute Kidney Injury | Documentation of AKI in clinical notes in the patient’s chart. |
| New Dialysis | Documentation of the initiation of hemodialysis, peritoneal dialysis, continuous veno-venous hemofiltration (CVVH), or continuous renal replacement therapy (CRRT) during hospital admission, in the patient’s chart. |
| Myocardial Infarction | Documentation of a new myocardial infarction in clinical notes in the patient’s chart. |
| New Heart Failure | Documentation of new congestive heart failure in clinical notes in the patient’s chart. |
| Arrhythmia | Documentation of a new arrhythmia in clinical notes in the patient’s chart. |
| Rhabdomyolysis | Documentation of new rhabdomyolysis in clinical notes in the patient’s chart. |
| DKA | Documentation of diabetic ketoacidosis in clinical notes in the patient’s chart. |
| Thrombosis | Documentation of imaging study (CT angiogram or venous doppler ultrasound) that noted thrombosis in the patient’s chart. |
| Total LOS | Total time from admission to discharge, death, or final chart review for one continuous admission. |
