# Supplementary material for: Obesity is independently associated with septic shock, renal complications, and mortality in a multiracial patient cohort hospitalized with COVID-19
Source: PLoS One. 2021 Aug 12;16(8):e0255811. doi: 10.1371/journal.pone.0255811 (PMC8360607; doi:10.1371/journal.pone.0255811)
Supplement: S3 Table — (DOCX) [file pone.0255811.s003.docx]

**S3a-d Table.** **Univariable logistic regression of BMI and relevant outcomes including a) intubation, b) mortality, c) septic shock, d) renal replacement therapy.**

| 1. **Intubation Univariable Logistic Regression Results** | | | | | |
| --- | --- | --- | --- | --- | --- |
|  | **No Intubation**  **(N=740)** | **Intubation**  **(N=278)** | **Odds Ratio** | **Confidence Interval** | **P-Value** |
| Age, median [IQR] | 64.0 [51.0-77.0] | 63.0 [52.0-72.0] | 0.99 | 0.98-1.00 | 0.058 |
| Male, (n%) | 411 (55.5) | 186 (66.9) | 1.62 | 1.21-2.16 | 0.001 |
| BMI, median [IQR] | 28.3 [24.8-32.7] | 29.5 [25.7-34.1] | 1.04 | 1.02-1.06 | <0.001 |
| CatBMI, n(%) |  |  |  |  |  |
| 18.5-24.9 (Reference) | 174 (23.5) | 53 (19.1) |  |  |  |
| <18.5 | 22 (3.0) | 3 (1.1) | 0.45 | 0.13-1.55 | 0.206 |
| 25-29.9 | 256 (34.6) | 90 (32.4) | 1.15 | 0.78-1.71 | 0.471 |
| 30-39.9 | 239 (32.3) | 101 (36.3) | 1.39 | 0.94-2.04 | 0.096 |
| ≥40 | 49 (6.6) | 31 (11.2) | 2.08 | 1.20-3.58 | 0.009 |
| Race, n(%) |  |  |  |  |  |
| White (Reference) | 179 (24.2) | 65 (23.4) |  |  |  |
| Asian | 13 (1.8) | 8 (2.9) | 1.69 | 0.67-4.28 | 0.264 |
| Black | 162 (21.9) | 72 (25.9) | 1.22 | 0.82-1.82 | 0.319 |
| Not Specified | 122 (16.5) | 49 (17.6) | 1.11 | 0.71-1.71 | 0.651 |
| Other | 264 (35.7) | 84 (30.2) | 0.88 | 0.60-1.28 | 0.490 |
| Patient Ethnicity, n(%) |  |  |  |  |  |
| Not Hispanic (Reference) | 201 (27.2) | 84 (30.2) |  |  |  |
| Hispanic | 396 (53.5) | 141 (50.7) | 0.85 | 0.62-1.17 | 0.325 |
| Not Specified | 143 (19.3) | 53 (19.1) | 0.89 | 0.59-1.33 | 0.561 |
| CAD, (n%) | 106 (14.3) | 36 (12.9) | 0.89 | 0.59-1.34 | 0.573 |
| HF, (n%) | 95 (12.8) | 30 (10.8) | 0.82 | 0.53-1.27 | 0.376 |
| CVA, (n%) | 67 (9.1) | 20 (7.2) | 0.78 | 0.46-1.31 | 0.345 |
| Diabetes, (n%) | 289 (39.1) | 119 (42.8) | 1.17 | 0.88-1.54 | 0.277 |
| HTN, (n%) | 457 (61.8) | 189 (68.0) | 1.32 | 0.98-1.76 | 0.066 |
| HLD, (n%) | 270 (36.5) | 100 (36.0) | 0.98 | 0.73-1.30 | 0.879 |
| COPD, (n%) | 54 (7.3) | 18 (6.5) | 0.88 | 0.51-1.53 | 0.649 |
| Asthma, (n%) | 73 (9.9) | 32 (11.5) | 1.19 | 0.77-1.85 | 0.442 |
| Renal Disease (Any), (n%) | 125 (16.9) | 34 (12.2) | 0.69 | 0.46-1.03 | 0.069 |
| Cancer, (n%) | 53 (7.2) | 15 (5.4) | 0.74 | 0.41-1.33 | 0.316 |
| Statins, (n%) | 294 (39.7) | 108 (38.8) | 0.96 | 0.73-1.28 | 0.798 |

| 1. **Mortality Univariable Logistic Regression Results** | | | | | |
| --- | --- | --- | --- | --- | --- |
|  | **No Mortality**  **(N=771)** | **Mortality**  **(N=239)** | **Odds Ratio** | **Confidence Interval** | **P-Value** |
| Age, median [IQR] | 61.0 [49.5-72.0] | 74.0 [64.0-84.0] | 1.05 | 1.04-1.06 | <0.001 |
| Male, (n%) | 443 (57.5) | 151 (63.2) | 1.27 | 0.94-1.71 | 0.117 |
| BMI, median [IQR] | 28.7 [25.3-33.1] | 28.3 [24.0-32.8] | 0.99 | 0.97-1.01 | 0.392 |
| CatBMI, n(%) |  |  |  |  |  |
| 18.5-24.9 (Reference) | 150 (20.1) | 71 (29.7) |  |  |  |
| <18.5 | 19 (2.5) | 6 (2.5) | 0.69 | 0.26-1.80 | 0.448 |
| 25-29.9 | 271 (35.1) | 72 (30.1) | 0.58 | 0.40-0.85 | 0.005 |
| 30-39.9 | 268 (34.8) | 69 (28.9) | 0.56 | 0.38-0.83 | 0.003 |
| ≥40 | 58 (7.5) | 21 (8.8) | 0.79 | 0.45-1.40 | 0.421 |
| Race, n(%) |  |  |  |  |  |
| White (Reference) | 184 (23.9) | 59 (24.7) |  |  |  |
| Asian | 14 (1.8) | 7 (2.9) | 1.56 | 0.60-4.05 | 0.361 |
| Black | 181 (23.5) | 51 (21.3) | 0.88 | 0.57-1.35 | 0.553 |
| Not Specified | 122 (15.8) | 47 (19.7) | 1.20 | 0.77-1.88 | 0.420 |
| Other | 270 (35.0) | 75 (31.4) | 0.87 | 0.59-1.28 | 0.470 |
| Patient Ethnicity, n(%) |  |  |  |  |  |
| Not Hispanic (Reference) | 216 (28.0) | 67 (28.0) |  |  |  |
| Hispanic | 405 (52.5) | 128 (53.6) | 1.02 | 0.73-1.43 | 0.914 |
| Not Specified | 150 (19.5) | 44 (18.4) | 0.95 | 0.61-1.46 | 0.801 |
| CAD, (n%) | 86 (11.2) | 53 (22.2) | 2.27 | 1.55-3.31 | <0.001 |
| HF, (n%) | 80 (10.4) | 45 (18.8) | 2.00 | 1.35-2.98 | 0.001 |
| CVA, (n%) | 51 (6.6) | 36 (15.1) | 2.50 | 1.59-3.94 | <0.001 |
| Diabetes, (n%) | 276 (35.8) | 127 (53.1) | 2.03 | 1.52-2.73 | <0.001 |
| HTN, (n%) | 444 (57.6) | 196 (82.0) | 3.36 | 2.34-4.81 | <0.001 |
| HLD, (n%) | 260 (33.7) | 106 (44.4) | 1.57 | 1.17-2.11 | 0.003 |
| COPD, (n%) | 43 (5.6) | 29 (12.1) | 2.34 | 1.42-3.84 | 0.001 |
| Asthma, (n%) | 92 (11.9) | 13 (5.4) | 0.42 | 0.23-0.77 | 0.005 |
| Renal Disease (Any), (n%) | 106 (13.7) | 51 (21.3) | 1.70 | 1.17-2.47 | 0.005 |
| Cancer, (n%) | 43 (5.6) | 24 (10.0) | 1.89 | 1.12-3.19 | 0.017 |
| Statins, (n%) | 280 (36.3) | 116 (48.5) | 1.65 | 1.23-2.22 | 0.001 |

| 1. **Septic Shock Univariable Logistic Regression Results** | | | | | |
| --- | --- | --- | --- | --- | --- |
|  | **No Septic Shock**  **(N=787)** | **Septic Shock**  **(N231** | **Odds Ratio** | **Confidence Interval** | **P-Value** |
| Age, median [IQR] | 64.0 [51.0-76.0] | 64.0 [53.5-72.0] | 1.00 | 1.00-1.01 | 0.872 |
| Male, (n%) | 447 (56.8) | 150 (64.9) | 1.41 | 1.04-1.91 | 0.028 |
| BMI, median [IQR] | 28.2 [24.8-32.7] | 29.7 [26.1-34.6] | 1.03 | 1.01-1.06 | 0.001 |
| CatBMI, n(%) |  |  |  |  |  |
| 18.5-24.9 (Reference) | 190 (24.1) | 37 (16.0) |  |  |  |
| <18.5 | 20 (2.5) | 5 (2.2) | 1.28 | 0.45-3.64 | 0.638 |
| 25-29.9 | 270 (34.3) | 76 (32.9) | 1.45 | 0.94-2.23 | 0.097 |
| 30-39.9 | 252 (32.0) | 88 (38.1) | 1.79 | 1.17-2.75 | 0.007 |
| ≥40 | 55 (7.0) | 25 (10.8) | 2.33 | 1.29-4.21 | 0.005 |
| Race, n(%) |  |  |  |  |  |
| White (Reference) | 195 (24.8) | 49 (21.2) |  |  |  |
| Asian | 12 (1.5) | 9 (3.9) | 2.98 | 1.19-7.48 | 0.020 |
| Black | 171 (21.7) | 63 (27.3) | 1.47 | 0.96-2.25 | 0.078 |
| Not Specified | 126 (16.0) | 45 (19.5) | 1.42 | 0.89-2.26 | 0.136 |
| Other | 283 (36.0) | 65 (28.1) | 0.91 | 0.60-1.38 | 0.670 |
| Patient Ethnicity, n(%) |  |  |  |  |  |
| Not Hispanic (Reference) | 211 (26.8) | 74 (32.0) |  |  |  |
| Hispanic | 431 (54.8) | 106 (45.9) | 0.70 | 0.50-0.98 | 0.041 |
| Not Specified | 145 (18.4) | 51 (22.1) | 1.00 | 0.66-1.52 | 0.989 |
| CAD, (n%) | 111 (14.1) | 31 (13.4) | 0.94 | 0.62-1.45 | 0.792 |
| HF, (n%) | 97 (12.3) | 28 (12.1) | 0.98 | 0.63-1.54 | 0.934 |
| CVA, (n%) | 71 (9.0) | 16 (6.9) | 0.75 | 0.43-1.32 | 0.318 |
| Diabetes, (n%) | 306 (38.9) | 102 (44.2) | 1.24 | 0.92-1.67 | 0.151 |
| HTN, (n%) | 484 (61.5) | 162 (70.1) | 1.47 | 1.07-2.02 | 0.017 |
| HLD, (n%) | 283 (36.0) | 87 (37.7) | 1.08 | 0.79-1.46 | 0.636 |
| COPD, (n%) | 59 (7.5) | 13 (5.6) | 0.74 | 0.40-1.37 | 0.332 |
| Asthma, (n%) | 84 (10.7) | 21 (9.1) | 0.84 | 0.51-1.38 | 0.487 |
| Renal Disease (Any), (n%) | 132 (16.8) | 27 (11.7) | 0.66 | 0.42-1.02 | 0.063 |
| Cancer, (n%) | 53 (6.7) | 15 (6.5) | 0.96 | 0.53-1.74 | 0.897 |
| Statins, (n%) | 309 (39.3) | 93 (40.3) | 1.04 | 0.77-1.41 | 0.785 |

| 1. **Renal Replacement Therapy Univariable Logistic Regression Results** | | | | | |
| --- | --- | --- | --- | --- | --- |
|  | **No RTT**  **(N=855)** | **RTT**  **(N=92)** | **Odds Ratio** | **Confidence Interval** | **P-Value** |
| Age, median [IQR] | 64.0 [52.0-76.0] | 61.0 [52.0-71.0] | 0.99 | 0.98-1.00 | 0.095 |
| Male, (n%) | 484 (56.6) | 70 (76.1) | 2.44 | 1.48-4.01 | <0.001 |
| BMI, median [IQR] | 28.5 [25.1-32.8] | 32.6 [27.4-36.6] | 1.06 | 1.03-1.09 | <0.001 |
| CatBMI, n(%) |  |  |  |  |  |
| 18.5-24.9 (Reference) | 194 (22.7) | 10 (10.9) |  |  |  |
| <18.5 | 18 (2.1) | 1 (1.1) | 1.08 | 0.13-8.90 | 0.945 |
| 25-29.9 | 302 (35.3) | 23 (25.0) | 1.48 | 0.69-3.17 | 0.317 |
| 30-39.9 | 277 (32.4) | 43 (46.7) | 3.01 | 1.48-6.14 | 0.002 |
| ≥40 | 64 (7.5) | 15 (16.3) | 4.55 | 1.95-10.62 | <0.001 |
| Race, n(%) |  |  |  |  |  |
| White (Reference) | 212 (24.8) | 16 (17.4) |  |  |  |
| Asian | 14 (1.6) | 5 (5.4) | 4.73 | 1.51-14.80 | 0.008 |
| Black | 178 (20.8) | 33 (35.9) | 2.46 | 1.31-4.61 | 0.005 |
| Not Specified | 147 (17.2) | 14 (15.2) | 1.26 | 0.60-2.66 | 0.542 |
| Other | 304 (35.6) | 24 (26.1) | 1.05 | 0.54-2.02 | 0.893 |
| Patient Ethnicity, n(%) |  |  |  |  |  |
| Not Hispanic (Reference) | 226 (26.4) | 38 (41.3) |  |  |  |
| Hispanic | 462 (54.0) | 40 (43.5) | 0.51 | 0.32-0.83 | 0.006 |
| Not Specified | 167 (19.5) | 14 (15.2) | 0.50 | 0.26-0.95 | 0.034 |
| CAD, (n%) | 116 (13.6) | 9 (9.8) | 0.69 | 0.34-1.41 | 0.311 |
| HF, (n%) | 94 (11.0) | 9 (9.8) | 0.88 | 0.43-1.80 | 0.723 |
| CVA, (n%) | 74 (8.7) | 7 (7.6) | 0.87 | 0.39-1.95 | 0.733 |
| Diabetes, (n%) | 325 (38.0) | 39 (42.4) | 1.20 | 0.78-1.86 | 0.412 |
| HTN, (n%) | 528 (61.8) | 61 (66.3) | 1.22 | 0.77-1.92 | 0.393 |
| HLD, (n%) | 305 (35.7) | 31 (33.7) | 0.92 | 0.58-1.44 | 0.707 |
| COPD, (n%) | 61 (7.1) | 7 (7.6) | 1.07 | 0.48-2.42 | 0.867 |
| Asthma, (n%) | 90 (10.5) | 9 (9.8) | 0.92 | 0.45-1.90 | 0.825 |
| Renal Disease (CKD Only), (n%) | 77 (9.0) | 12 (13.0) | 1.52 | 0.79-2.90 | 0.210 |
| Cancer, (n%) | 59 (6.9) | 6 (6.5) | 0.94 | 0.39-2.24 | 0.891 |
| Statins, (n%) | 321 (37.5) | 34 (37.0) | 0.98 | 0.62-1.52 | 0.912 |
